# The introduction of the practice nurse mental health in general practices in the Netherlands: effects on number of diagnoses of chronic and acute alcohol abuse

**DOI:** 10.1186/s12875-019-0938-3

**Published:** 2019-04-02

**Authors:** L. Abidi, A. Oenema, P. Verhaak, F. E. S. Tan, D. van de Mheen

**Affiliations:** 1 Department of Health Promotion, Care and Public Health Research Institute, Maastricht University, PO Box 616, 6200 MD Maastricht, the Netherlands; 2 NIVEL, Netherlands Institute of Health Services Research, Utrecht, the Netherlands; 3 Tranzo, Tilburg University, School of Social and Behavioral Sciences, Tilburg, the Netherlands; 4 Department of Family Practice, Groningen University, University Medical Center Groningen, Groningen, the Netherlands; 5 Department of Statistics and Methodology, Care and Public Health Research Institute, Maastricht University, Maastricht, the Netherlands

**Keywords:** Alcohol abuse, Practice nurse mental health, Collaborative care, Nurse-led care

## Abstract

**Background:**

Since 2008 mental health practice nurses have been gradually introduced in general practices in the Netherlands as part of health policy aiming to improve early identification and treatment of mental health problems in primary care. This study aims to investigate the effect of the introduction of the practice nurse mental health in general practices in the Netherlands on the number of diagnoses of chronic and acute alcohol abuse.

**Methods:**

The Netherlands Institute for Health Services Research (NIVEL) retrieved data of a representative sample of general practices (*n* = 155) for this study. Data were aligned at the starting point of the implementation of the PN-MH to compare the practices on our outcome measures after implementation of the PN-MH. Multilevel regression analyses were conducted to investigate differences in average number of chronic and acute alcohol abuse diagnoses between practices with a practice nurse mental health and control practices (without a practice nurse mental health and without a primary care psychologists).

**Results:**

A significant decrease over time of chronic alcohol abuse diagnoses was observed (ß = -.52, *p* < 0.05) as well as a significant decrease over time of acute alcohol abuse diagnoses (ß = -.06, *p* < 0.05). After adjustment for multiple comparisons, no significant differences were found between practices that implemented a practice nurse mental health or only have a primary care psychologist and control practices. Practices that implemented a practice nurse mental health and have a primary care psychologist, had a higher mean of chronic and acute alcohol abuse diagnoses than control practices during all periods, but the differences between these groups were not statistically significant.

**Conclusions:**

Based on the results of this study it seems that the introduction of practice nurses mental health in general practices is not associated with increased diagnoses of chronic or acute alcohol abuse. Potential explanations are barriers experienced by practice nurses to addressing alcohol use with patients and prioritization of other mental health issues over alcohol abuse. In order to improve the management of alcohol abuse by practice nurses, more research is needed on how practice nurses can be involved in diagnosing and treatment of patients with alcohol abuse.

## Background

Alcohol abuse is associated with considerable morbidity and mortality and contributes significantly to the global burden of disease [1] as well as to enormous societal costs [2]. Alcohol abuse (or alcohol use disorder) is defined as problematic alcohol use where at least two of the 11 criteria specified in the DSM-5 are met within 12-months [3]. Prevalence estimates of alcohol abuse in the Netherlands range from 1 to 10.4% depending on the severity of the alcohol use and related problems [4, 5].

In the Netherlands, the general practitioner (GP) is the first doctor to be contacted when people have health problems and almost all Dutch residents are registered with a general practice. General practice, therefore, offers a good opportunity for early detection and delivery of brief interventions targeting a wide proportion of the population. However, alcohol problems are frequently not discussed or recognized in primary health care [6–8]. In general, lack of time and lack of access to necessary (low-threshold) services are mentioned by GPs as important barriers for discussing alcohol use and delivering brief interventions [7, 9].

As primary care provides highly accessible services and secondary care is relatively expensive, recent changes in the Dutch healthcare system were aimed at a more eminent role for mental health care within primary care and in particular in general practices. To accommodate GPs in their larger role in providing mental health care, from 2008 the Practice Nurse Mental Health (PN-MH) has been introduced in general practices [10, 11]. In order to encourage the shift from secondary to primary mental health care and to save costs, the Ministry of Health, Wellfare and Sports, decided to provide more financial means in 2012, 2013 and 2014 to stimulate the deployment of the PN-MH in general practices. In 2013, the maximum deployment hours of a PN-MH per practice have been doubled from 4.5 h to 9 h a week and as of January 1st 2014 GPs received extra funding enabling them to work with a PN-MH. Consequently, in 2011 34% of all GP’s in the Netherlands deployed a PN-MH in their practice, in 2012 50% of all general practices employed a PN-MH and in 2015 the percentages of general practices that deployed a PN-MH rose to 85% [12].

The PN-MH provides support in general practice care to all patients with psychological, psychosocial or psychosomatic symptoms, while working under supervision of the GP (Trimbos-institute, 2014a). The role of the PN-MH is rather new but a function and competence profile describes that the tasks of the PN-MH often include diagnostic clarification, screening, referring to other mental health caregivers and providing accessible mental health consultation and brief advice or short-term treatment based on motivational interviewing or psycho-education for patients with early signs of psychological disorders or social problems [13]. As the PN-MH provides mental health consultation and brief advice or short-term treatment within general practice, the PN-MH can be easily reached and patients can be easily referred (i.e. low-threshold service). The presence of a PN-MH could also provide an opportunity for more attention to early detection and treatment of patients with alcohol abuse. Therefore, employing a PN-MH into general practices might lead to an increase in detection and diagnoses of patients with chronic or acute alcohol abuse in general practices. Moreover, GPs might increase their willingness to talk about alcohol use with their patients as they can refer patients to a PN-MH within their own practice. This, therefore, might be a solution to the previously mentioned barrier of lack of access to low-threshold mental health care.

Many practices had already implemented a primary care psychologist (PCP) before the introduction of the PN-MH, who focuses on diagnosis and treatment of patients with more severe psychological symptoms or a possible full disorder [14]. In the Dutch reimbursement system, care from the PN-MH falls within the basic health insurance coverage and is fully reimbursed. Care from the PCP, however, falls within the part of the insurance system for which patients have to pay the first € 335,- (in 2013) of costs themselves (i.e. deductible). Therefore, care from the PN-MH is more low-threshold and less expensive.

The current study aims to investigate the effect of the introduction of the PN-MH in general practices in the Netherlands on the number of diagnoses of alcohol abuse. Up till now, these effects are unknown. As previous studies have shown [6, 7, 15] that a lack of time and a lack of a low-threshold referral options for GPs are important barriers for discussing alcohol use and delivering brief interventions, we expect that practices that have employed a PN-MH will contribute to higher detection of alcohol abuse compared to control practices (practices without a PN-MH and PCP).

## Methods

### Design

This is an observational study using data collected by The Netherlands Institute for Health Services Research (NIVEL). The NIVEL Primary Care Database contains routinely kept electronic medical record data from practices, equally distributed through the Netherlands. All GP-patient contacts are recorded in this database with information about diagnosis, prescriptions and activities, as well as information about which practices have deployed a PN-MH. We requested electronic medical records data about alcohol abuse diagnoses from a representative sample of general practices from 2011 to 2013. Data of 187 practices in 2011, 2012, 2013 was available of which 155 practices were included in the analyses. In this time frame practices could voluntarily implement the PN-MH. Of the 155 practices, 86 practices had implemented the PN-MH in the course of this three-year time period. We have further made a distinction between practices who have implemented a PN-MH and practices that already had a PCP and additionally implemented a PN-MH. As a result, a total of 46 practices are practices without a PN-MH and PCP, 23 practices have only a PCP, 56 practices have only a PN-MH and 30 practices have both a PN-MH and PCP. The main outcome measure was the number of diagnoses of alcohol abuse.

As the implementation of the PN-MH in general practices occurred gradually over the years (2011–2013), data was aligned at the starting point of the implementation of the PN-MH to be able to compare the practices on our outcome measures post-implementation. This means that for each practice the date of implementation of the PN-MH was taken as the starting point in the study and that the follow-up period length differs between practices.

Because the number of diagnoses per month provided us with 36 time-points and a possibly too fragmented picture, we aggregate 36 time-points to six periods of six months (three years in total). Period-prevalence of alcohol abuse diagnoses were therefore calculated over six periods of six months.

### Measurements

The Netherlands Institute for Health Services Research (NIVEL) Primary Care Database has been used to subtract routinely recorded data from a representative sample of Dutch general practices and patients. We used data derived from routine electronic medical records of GP practices. Electronic medical records contain information about diagnoses as well as demographic information (gender and age).

For the registration of diagnoses, GPs use the International Classification of Primary Care (ICPC) [16]. The ICPC has been developed to document systematically the episodes of care. In the ICPC, two codes concern alcohol: P15 — Chronic Alcohol Abuse and P16 — Acute Alcohol Abuse. In our study, we analyzed the number of diagnoses in both categories Chronic alcohol abuse (ICPC: P15) and Acute alcohol abuse (ICPC: P16).

Chronic alcohol abuse is defined as problematic alcohol use where at least two of the 11 criteria specified for the disorder in the DSM-5 are met within 12-months. Criteria are e.g. 1) ending up drinking more, or longer, than intended, 2) Spending a lot of time drinking, or being sick or getting over other aftereffects, 3) Found that drinking—or being sick from drinking—often interfered with taking care of your home or family, or caused job troubles, etc.. Acute alcohol abuse is defined as excessive alcohol consumption in a short period of time in which the symptoms are dose-dependent and can include disorientation, elated mood, labile mood, sexual and/or aggressive disinhibition and impairment of speech (dysarthria) [17]. Dutch GPs use these definitions for diagnosing chronic or acute alcohol abuse based on the guidelines from the Dutch College of General Practitioners (NHG) [17].

### Analyses

Descriptive analyses were used to describe the number of patients with alcohol problem diagnoses (P15 or P16), and to describe the composition of the GP practices.

To investigate differences between groups in average number of diagnoses (P15 or P16), data were analyzed with multilevel linear regression analyses using a random intercept model [18]. A two-level hierarchical structure was used: general practices measured over time as the first level and with practices as a random factor (second level). We determined the covariance structure of the random effects in our model, by starting with an unstructured matrix. By using the Likelihood Ratio we determined the best fitting model (which included all fixed effects) to be a random intercept model. First, a regression model with time as a continuous predictor was conducted to investigate overall time effects. To investigate whether practices with a PN-MH differ from the comparison-group (i.e. practices without a PN-MH and without PCP) over the different time-periods, dummy-variables were created for the six time-periods and for the four groups. Time x group interaction terms between all time-dummies and group-dummies were included in the model. The model was adjusted for general practitioners’ Full-Time Equivalent (FTE) per practice and number of registered patients. The independent variable was group (control practices without PN-MH and PCP, practices with a PCP, practices with a PN-MH and practices with both a PCP and a PN-MH). All three groups were compared with control practices. The significance level was set at *P <* 0.05/3 = 0.016, adjusted for three comparisons according to the Bonferroni method. The outcome variables are amount of diagnoses of chronic alcohol abuse, amount of diagnoses of acute alcohol abuse. To account for potential differences in number of diagnoses in the pre-implementation period between the three groups, the analyses were repeated with number of diagnoses in the pre-implementation period as an additional covariate. These analyses could only be conducted among the practices that introduced the PN-MH after the first six-month period (*n* = 99), and therefore, these analyses were considered as a sensitivity test. Data was aligned at the starting point of the implementation of the PN-MH which led to missing values by design at follow-up periods. It can be assumed that the missing values of the outcome are missing at random (MAR). Rather than analyzing complete cases only, potentially biasing estimates, parameter estimates with Full Information Maximum Likelihood, will provide unbiased estimates [18]. Due to missing observations in the covariate ‘Full-Time Equivalent (FTE) per practice’ in 32 practices, complete data was available and analyzed for 155 practices of the 187 practices (83% of the sample). The missing observations are assumed to be independent of the outcome variable. All analyses were performed using the software program Statistical Package for Social Sciences (SPSS 23).

### Ethical approval

NIVEL Primary Care Database collects anonymized data for research. GPs participating in NIVEL Primary Care Database brief patients by leaflets and posters about the anonymous use of their data. Patients are offered an opportunity for opting out. According to Dutch legislation neither obtaining informed consent nor approval by a medical ethics committee was obliged for database studies without direct patient involvement. The NIVEL Primary Care Database is registered with the Dutch Data Protection Authority. As Dutch law allows the use of anonymized electronic medical records for research purposes under certain conditions, we did not need informed consent or approval by a medical ethics committee for this study (Dutch Civil Law, Article 7:458).

## Results

### Practice characteristics

Data were obtained from a total of 155 general practices, of which 46 practices are control practices (without a PN-MH and PCP), 23 practices only have a PCP, 56 practices only have a PN-MH and 30 practices have both a PN-MH and PCP. Overall, general practitioners have an average workload of 2.35 Full-time equivalent (FTE) (Table 1).

**Table 1:** Practice characteristics

	Practices without PN-MH and PCP	Practices with PCP	Practices with PN-MH	Practices with PCP & PN-MH
General practitioners, N	46	23	56	30
Full-time equivalent (FTE) general practitioners (mean)	2.06	2.57	2.20	2.56
Patient population 2011 (mean^a^)	3557.8	4145,4	3442.0	3929.0
Patient population 2012 (mean)	3755.8	4471,0	3561.5	4584.5
Patient population 2013 (mean)	3769.1	4447,4	3664.9	4810.6
Overall number of diagnoses (P15)	1843	992	1612	1138
Overall number of diagnoses (P16)	358	149	363	215

^a^population means were calculated for the respective year

### Diagnoses

Table 2 presents the absolute number of diagnoses of chronic alcohol abuse and acute alcohol abuse in Dutch general practices per six month periods. In the 155 practices in the analysis, 5.535 episode diagnoses of chronic alcohol abuse or acute alcohol abuse were made in 3 years of time.

**Table 2:** Absolute numbers of chronic and acute alcohol abuse diagnoses, means (SD)

	^a^Period 1 (0–6 months)	Period 2 (7–12 months)	Period 3 (13–18 months)	Period 4 (19–24 months)	Period 5 (25–30 months)	Period 6 (31–36 months)
Diagnoses of chronic alcohol abuse						
Control (*N* = 46)	323, 7.0 (4.8)	274, 6.0 (6.2)	241, 5.2 (4.6)	282, 6.1 (5.5)	205, 4.5 (3.7)	201, 4.7 (3.8)
PCP (*N* = 23)	248, 10.8 (8.4)	168, 7.3 (7.1)	179, 7.8 (5.5)	131, 5.7 (4.6)	127, 5.8 (4.3)	139, 6.3 (4.5)
PN-MH (*N* = 56)	313, 5.6 (4.9)	253, 4.9 (5.2)	201, 5.0 (6.0)	208, 5.2 (6.6)	118, 3.8 (3.9)	75, 2.8 (2.9)
PCP/PN-MH (*N* = 30)	251, 8.4 (7.2)	256, 9.1 (7.5)	142, 7.1 (9.0)	143, 7.2 (5.7)	119, 6.6 (5.4)	61, 4.4 (3.7)
Total (*N* = 155)	1135	951	763	764	569	476
Diagnoses of acute alcohol abuse						
Control (*N* = 46)	43, 0.9 (1.4)	62, 1.3 (1.7)	37, 0.8 (1.2)	63, 1.4 (1.6)	50, 1.1 (1.1)	42, 1.0 (1.1)
PCP (*N* = 23)	24, 1.0 (1.0)	26, 1.1 (1.6)	28, 1.2 (1.4)	21, 0.9 (1.6)	26, 1.2 (1.3)	24, 1.1 (1.1)
PN-MH (*N* = 56)	61, 1.1 (1.8)	66, 1.3 (1.8)	40, 1.0 (1.7)	47, 1.2 (1.7)	22, 0.7 (0.8)	19, 0.7 (1.3)
PCP/PN-MH (*N* = 30)	47, 1.6 (2.5)	44, 1.6 (2.0)	34, 1.7 (2.5)	26, 1.3 (1.9)	16, 1.0 (1.5)	9, 0.6 (0.9)
Total (*N* = 155)	175	198	139	157	114	94
Total	5.535 diagnoses					

^a^Different periods indicate the number of months after implementation of the PN-MH

**Table 3:** Multilevel linear regression analyses: group, time and interaction effects on diagnoses of chronic and acute alcohol abuse

	Diagnoses of chronic alcohol abuse		Diagnoses of acute alcohol abuse and intoxication	
	β (SE)	*P*	β (SE)	*P*
Group (control)				
PCP	0.85 (0.75)	.26	−0.12 (0.23)	.60
PN-MH	−0.76 (0.70)	.28	0.01 (0.22)	.95
PCP/PN-MH	−0.13 (0.83)	.87	− 0.09 (0.26)	.72
±Time period (6)				
Period 1 (0–6 months)	2.40 (0.71)	.00*	−0.04 (0.21)	.84
Period 2 (7–12 months)	1.33 (0.63)	.04	0.37 (0.22)	.09
Period 3 (13–18 months)	0.65 (0.64)	.32	−0.17 (0.20)	.39
Period 4 (19–24 months)	1.60 (0.64)	.01*	0.41 (0.19)	.04
Period 5 (25–30 months)	−0.12 (1.22)	.76	0.13 (0.18)	.46
Interaction effects				
PCP/PN-MH * Period 1	0.60 (1.21)	.62	0.43 (0.37)	.24
PCP/PN-MH * Period 2	2.45 (1.13)	.03	0.01 (0.40)	.98
PCP/PN-MH * Period 3	0.96 (1.19)	.42	0.75 (0.37)	.04
PCP/PN-MH * Period 4	0.19 (1.20)	.87	−0.19 (0.37)	.60
PCP/PN-MH * Period 5	1.66 (0.79)	.03	−0.29 (0.34)	.40
PN-MH * Period 1	−0.62 (1.03)	.55	0.06 (0.31)	.84
PN-MH * Period 2	−0.23 (0.95)	.81	−0.10 (0.33)	.77
PN-MH * Period 3	0.93 (0.99)	.35	0.23 (0.31)	.46
PN-MH * Period 4	−0.06 (0.98)	.95	−0.19 (0.30)	.52
PN-MH * Period 5	0.70 (0.66)	.29	−0.25 (0.28)	.38
PCP * Period 1	2.25 (1.22)	.07	0.01 (0.36)	.98
PCP * Period 2	−0.16 (1.09)	.88	−0.32 (0.38)	.41
PCP * Period 3	0.99 (1.10)	.37	0.31 (0.34)	.36
PCP * Period 4	−1.87 (1.12)	.09	−0.52 (0.34)	.13
PCP * Period 5	−0.42 (0.69)	.54	−0.04 (0.30)	.88

*P < .016 (adjusted for multiple comparisons)

± = Reference period

Reference group for the interaction-terms is control group

ICC = 16.94

### Differences over time and between practices

Results show a significant time effect for chronic and acute alcohol abuse. From time period one through six, there was a significant decrease of chronic alcohol abuse diagnoses (ß = -.52, *p* < 0.05) as well as a significant decrease of acute alcohol abuse diagnoses (ß = -.06, *p* < 0.05).

**Fig. 1:**
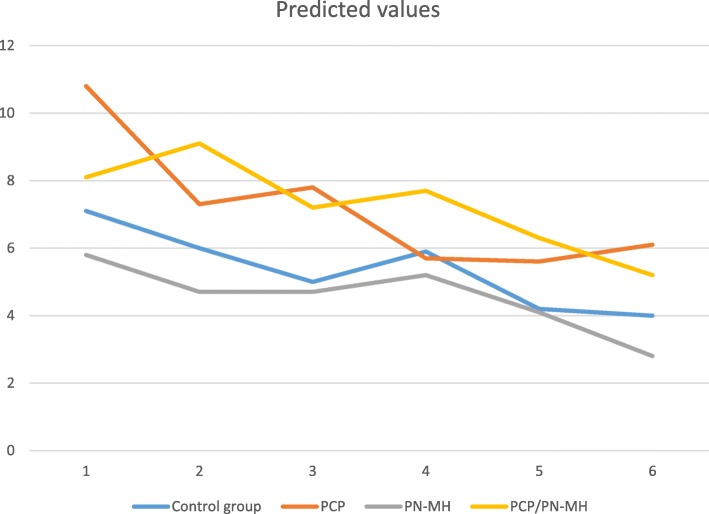
Predicted values (means) for chronic alcohol abuse diagnoses

Practices that had implemented the PN-MH had the lowest means of chronic alcohol abuse diagnoses during all time periods (Fig. 1), but there were no significant differences with control practices at any time period (Table 3). Practices that had implemented a PN-MH in addition to a PCP, had a higher mean of chronic alcohol abuse diagnoses than control practices, during all periods (Fig. 1), but the differences between these groups was not statistically significant at any time period. Even though the overall time trend shows a significant decrease over time, in the first year after the implementation of the PN-MH there was an increase in number of chronic alcohol abuse diagnoses in practices that implemented a PN-MH in addition to a PCP as compared to control practices, although this difference was not significant after adjustment for multiple comparisons (ß = 2.45, *p* > 0.016). Practices that solely have a PCP also showed non-significant differences in mean number of chronic alcohol abuse diagnoses compared to control practices.

**Fig. 2:**
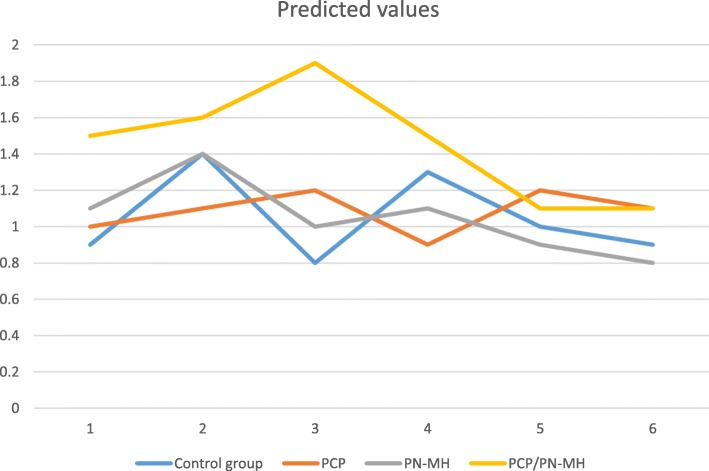
Predicted values (means) for acute alcohol abuse diagnoses

Regarding acute alcohol abuse diagnoses, in practices that have implemented the PN-MH or only have a PCP the average number of acute alcohol abuse diagnoses did not significantly differ from control practices in any of the time periods. Practices that implemented a PN-MH and that have a PCP show the highest means of acute alcohol abuse diagnoses during the first two years (Fig. 2), but there were no differences with the control group after adjustment for multiple comparisons (ß = -.75, *p* > 0.016).

### Sensitivity analyses

Sensitivity analyses with baseline data available of a subset of practices were conducted (41 control practices, 21 practices with a PCP, 24 practices with a PN-MH and 13 practices with both a PCP and a PN-MH) to control for pre-implementation variability in number of chronic or acute alcohol abuse diagnoses. The analyses were repeated using a random intercept model with number of diagnoses in the pre-implementation period as an additional covariate. Results show a significant decrease of chronic alcohol abuse diagnoses (ß = -.41, *p* < 0.05) while controlling for baseline number of chronic alcohol diagnoses. No significant decrease of acute alcohol abuse diagnoses was observed when controlling for baseline diagnoses (ß = -.02, *p* > 0.05). No significant differences with control practices at any time period were observed for both outcome variables.

## Discussion

This study investigated the effects of the introduction of the PN-MH on number of diagnoses of alcohol abuse. Results show that practices that have implemented the PN-MH did not significantly differ from control practices in mean number of chronic or acute alcohol abuse diagnoses, during any time period. Our results show a significant time effect indicating a decrease over time in both chronic and acute alcohol abuse diagnoses.

Based on the results of this study it seems that the PN-MH does not contribute to increased chronic or acute alcohol abuse diagnoses in general practices, even when accounting for differences in number of diagnoses in the pre-implementation period. These findings are in contrast to our expectations. One explanation for our findings is that PNs-MH experience similar barriers to addressing alcohol use and alcohol screening as reported by GPs. For instance, previous studies have shown that practice nurses find alcohol use a difficult subject to address and are concerned about possible negative reactions to lifestyle advice from patients [28]. It also appears that mostly patients with depression, neurasthenia, anxiety and stress are referred to the PN-MH, while patients with alcohol problems are less often referred to the PN-MH [13]. This suggest that in daily practice the service of the PN-MH is not as often utilized for patients with alcohol problems. In line with our findings, one previous study has shown that brief alcohol intervention delivery was insufficiently implemented by practice nurses in primary health care and resulted in low screening and brief intervention delivery rates [29]. These results indicate that in order to improve diagnosing and treatment of alcohol abuse by practice nurses in general practices, more research is needed into how practice nurses can be involved in diagnosing and counseling patients with alcohol abuse. Other factors such as health professionals’ own alcohol use and a higher prioritization of other mental health issues over alcohol might also play a role. Furthermore, the role of the PN-MH is rather new and the tasks of the PN-MH vary and encompass support to all patients with psychological, psychosocial or psychosomatic symptoms [13]. It might therefore be difficult to find effects on only one or two specific outcome measures. Acute alcohol abuse also has a low prevalence in general practice which makes it more challenging to detect changes.

It was expected that the cooperation between the PN-MH, PCP and GPs would lead to increased diagnoses of chronic and acute alcohol abuse. Previous studies have shown that multidisciplinary cooperation does influence quality of health care [19, 20]. The importance of multidisciplinary cooperation and integrating mental health care into primary care is supported by previous studies suggesting that when mental health care and general medical care providers work together to address both the physical and mental health needs of their patients’ access to treatment and quality of care improve [20]. It appears that consultations, shared medical records, systematic screening for mental health problems and regularly scheduled intervision and case reviews between care providers are often part of multidisciplinary cooperation. While it is not clear which of these elements are important more research is needed to investigate specific elements of care processes and cooperation between different care providers to understand what cooperation components lead to increased attention to diagnosing and treatment of alcohol abuse in general practices.

Our results also showed a time effect indicating an overall decrease in the amount of chronic alcohol abuse diagnoses in all time periods. One possible explanation for this result is a *saturation effect.* As increasing numbers of patients with psychological or social problems visit general practices [12] it may be the case that after a certain time period the number of new cases to be detected is lower. Furthermore, it is known that registrations of alcohol abuse diagnoses are not always complete [8, 27]. This hindrance among GPs to register diagnoses of alcohol abuse might be due to the associated stigma [25], and could play a role among PNs-MH as well. Therefore, it could be the case that alcohol use was actually discussed but recordings of diagnoses avoided and therefore missed in the registration systems. Future research should investigate this further by using both quantitative as well as qualitative research methods. In contrast to chronic alcohol abuse diagnoses, no significant decrease of acute alcohol abuse diagnoses was observed when controlling for baseline diagnoses. These results indicate a small (non-significant) average increase of acute alcohol abuse diagnoses from the pre-implementation period to the first post-implementation period followed by an average decrease in all subsequent periods. The decrease in chronic alcohol abuse as well as acute alcohol abuse diagnoses may be related to the interventional aspect of the PN-MH: a practice nurse typically has more time and is involved in treatment as well as referring patients to specialized care [10]. This may cause earlier treatment and recovery, and a consequential decrease in diagnoses. However, this should be further explored in future studies.

The WHO states that integrating mental health services into primary care leads to better treatment coverage, good health outcomes and cost effectiveness [22]. The results of our study indicate that just adding mental health services to care in general practices may not lead to improvements in detection and treatment of alcohol abuse and that more attention to this topic is needed to effectively improve this. It has to be noted, however, that our study covered the years 2011, 2012 and 2013. Since that time new health care innovations have been introduced in the Netherlands and the full potential of the introduction of the PN-MH may not have been reached yet. For instance, in 2014 in the Netherlands, the government made reforms to the mental health care system to decrease the substantial demands in secondary specialized mental health care. More support for the GP to treat minor mental health problems was made available, a new referral model for the GP and a new treatment model for the basic mental health care were implemented [26]. Consequently, more patients with psychological problems or symptoms such as anxiety or depression are seeking treatment within general practice: in the first six months of 2014 there was a 21% increase in consultations for psychological diagnoses compared to the first six months of 2013 [23]. It is currently still unclear how these reforms in general practices impacted on the role of the PN-MH and influenced diagnoses and treatment rates of alcohol abuse specifically. This needs to be investigated in future studies.

### Strengths and limitations

This study had strengths and limitations. This is the first study that investigates the effects of the introduction of the PN-MH in general practices on alcohol abuse diagnoses. The data used in this study are representative of the Dutch general practices and also representative of the Dutch population regarding age and gender [24]. Contrary to studies with a randomized controlled design, we were only able to analyze routinely collected data after the voluntary implementation of the PN-MH in general practices. This means that differences between groups or the lack of differences between groups may also have been influenced by other factors. Nevertheless, this naturalistic experimental study gives insight into what happens in practice after implementation of the PN-MH. Natural experimental studies are often recommended as a way of understanding the impact of policies when it is impossible to manipulate exposure to the intervention. Also, missing observations at post-test periods arose due to the design of the study. By assuming that there were no changed external influences during the studied time periods we considered the missing post-test observations to be missing at random (MAR). However, multilevel regression analysis offers appropriate ways to deal with missing data. In the analyses of the data we adjusted our model for confounding variables, by including working hours (i.e. GPs’ Full-Time Equivalent (FTE)) per practice and number of registered patients as covariates. However, data about opening hours of general practices or other possibly confounding variables were not available. Also, it is important to note that the investigated data are period prevalence data of diagnoses. This means that that the same patients may have had multiple diagnoses of alcohol abuse over time (e.g. in case of relapse). Data about incidence was not included in this study. Furthermore, it is important to note that our results are limited in generalizability to other countries as countries differ in health care systems, task roles of health professionals, reimbursement schemes and financial regulations regarding mental health in primary care.

## Conclusion

We conclude that the introduction of practice nurses mental health in general practices is not associated with increased diagnoses of chronic or acute alcohol abuse. This may be due to barriers experienced by practice nurses to addressing alcohol use with patients or prioritization of other mental health issues over alcohol abuse. In order to improve the management of alcohol abuse by practice nurses in general practices, more research is needed on how practice nurses can be involved in diagnosing and treatment of patients with alcohol abuse.

## Abbreviations

GP: General Practitioner
ICPC: International Classification of Primary Care
NIVEL: Netherlands Institute for Health Services Research
PCP: Primary Care Psychologist
PN-MH: Practice Nurse Mental Health
WHO: World Health Organisation
